# Presence of sst5TMD4, a truncated splice variant of the somatostatin receptor subtype 5, is associated to features of increased aggressiveness in pancreatic neuroendocrine tumors

**DOI:** 10.18632/oncotarget.6565

**Published:** 2015-12-11

**Authors:** Miguel Sampedro-Núñez, Raúl M. Luque, Ana M. Ramos-Levi, Manuel D. Gahete, Ana Serrano-Somavilla, Alicia Villa-Osaba, Magdalena Adrados, Alejandro Ibáñez-Costa, Elena Martín-Pérez, Michael D. Culler, Mónica Marazuela, Justo P. Castaño

**Affiliations:** ^1^ Department of Endocrinology and Nutrition, Hospital Universitario de la Princesa, Instituto de Investigación Princesa, Universidad Autónoma de Madrid, Madrid 28006, Spain; ^2^ Department of Cell Biology, Physiology and Immunology, University of Cordoba, Instituto Maimónides de Investigación Biomédica de Córdoba (IMIBIC), Hospital Universitario Reina Sofia, and CIBER Fisiopatología de la Obesidad y Nutrición, Córdoba 14014, Spain; ^3^ IPSEN Bioscience, Cambridge, Massachusetts 02142, USA

**Keywords:** neuroendocrine tumors, sst5TMD4, sst5TMD5, angiogenesis, gastroenteropancreatic neuroendocrine tumors

## Abstract

**Purpose:**

Gastroenteropancreatic neuroendocrine tumors (GEP-NETs) are rare and heterogeneous tumors, and their biological behavior is not well known. We studied the presence and potential functional roles of somatostatin receptors (sst1-5), focusing particularly on the truncated variants (sst5TMD4, sst5TMD5) and on their relationships with the angiogenic system (Ang/Tie-2 and VEGF) in human GEP-NETs.

**Experimental Design:**

We evaluated 42 tumor tissue samples (26 primary/16 metastatic) from 26 patients with GEP-NETs, and 30 non-tumoral tissues (26 from adjacent non-tumor regions and 4 from normal controls) from a single center. Expression of sst1-5, sst5TMD4, sst5TMD5, Ang1-2, Tie-2 and VEGF was analyzed using real-time qPCR, immunofluorescence and immunohistochemistry. Expression levels were associated with tumor characteristics and clinical outcomes. Functional role of sst5TMD4 was analyzed in GEP-NET cell lines.

**Results:**

sst1 exhibited the highest expression in GEP-NET, whilst sst2 was the most frequently observed sst-subtype (90.2%). Expression levels of sst1, sst2, sst3, sst5TMD4, and sst5TMD5 were significantly higher in tumor tissues compared to their adjacent non-tumoral tissue. Lymph-node metastases expressed higher levels of sst5TMD4 than in its corresponding primary tumor tissue. sst5TMD4 was also significantly higher in intestinal tumor tissues from patients with residual disease of intestinal origin compared to those with non-residual disease. Functional assays demonstrated that the presence of sst5TMD4 was associated to enhanced malignant features in GEP-NET cells. Angiogenic markers correlated positively with sst5TMD4, which was confirmed by immunohistochemical/fluorescence studies.

**Conclusions:**

sst5TMD4 is overexpressed in GEP-NETs and is associated to enhanced aggressiveness, suggesting its potential value as biomarker and target in GEP-NETs.

## INTRODUCTION

Neuroendocrine tumors (NETs) comprise a heterogeneous group of neoplasms derived from enterochromaffin epithelial cells, which retain many structural and functional features of normal endocrine cells, including production of chromogranin A (CgA), synaptophysin, and other peptides [1]. The majority of these tumors are of gastro-entero-pancreatic origin (GEP-NET) and, although they were initially believed to be uncommon neoplasms, their incidence and prevalence is increasing, and not only because of improved imaging techniques [2].

Unlike other malignancies, the natural history of NETs is significantly variable and difficult to predict. Most patients with well-differentiated GEP-NETs, even if metastatic disease is present at diagnosis, may present with a relatively indolent course, whilst others may progress rapidly, with median survival duration ranging from 5 to 56 months in advanced stage disease [2]. To date, there are very few biomarkers of prognosis, which could be useful to assess prognosis and select patients whose disease may progress rapidly or who may benefit from early initiation of therapy [3].

Although the first therapeutic option for GEP-NETs is the surgical approach, complete cure is not possible in many cases, and development of systemic medical treatments has gained scientific and clinical interest over the past recent years. In this setting, synthetic somatostatin analogues (SSAs) have emerged as a successful tool for the management of neuroendocrine diseases [4, 5]. SSAs inhibit hormonal secretion by binding to ssts, and thus provide relief of symptoms in patients with functional NETs. Additionally, they exert antitumor effects; this was confirmed by the results of the PROMID study [6], which reported a significant increase in time to tumor progression in functionally active and inactive tumors; and in a more evident way, in the recent CLARINET study, which further reported an increase in median progression-free survival in SSA-treated patients [7].

SSAs exert their biological actions by binding to a family of G protein-coupled, seven transmembrane-spanning somatostatin receptors (sst1-sst5) in neuroendocrine cells, which, depending on the tumor type and the specific set of receptors involved, lead to decreased hormonal secretion, decreased growth and mitotic rates, increased apoptosis, and/or inhibition of cell signaling and protein synthesis, including inhibition of production and secretion of various angiogenic factors [8–11]. In mammals, ssts are encoded by five separate intronless genes (SSTR1-SSTR5), which have been classically considered to give rise to five different somatostatin receptors, named sst1 through sst5, plus, in mouse, a carboxyterminal spliced variant of sst2, named sst2B. However, recent studies from our group have unveiled the existence of new truncated, albeit functional sst5 variants, generated by non-canonical splicing, which in humans bear 5 and 4 transmembrane domains (instead of the usual 7), and are therefore referred to as sst5TMD4 and sst5TMD5 [12, 13].

As sst subtypes represent obligatory mediators of SSA actions, variability in the sst1-5 expression profile in NET cells has been reasonably suggested as a potential predictive factor for SSA response. In fact, antiproliferative effects of SSAs have been associated to their affinity for sst2 [14, 15]; and conversely, the tachyphylaxis that eventually develops in some cases during long-term management of NETs has been attributed to a possible loss of sst2 availability following receptor internalization and degradation [16]. Moreover, since antisecretory and antiproliferative effects occur at different time-windows, involvement of different receptors and/or molecular mechanisms has been also proposed [17]. For instance, the presence of the truncated variant sst5TMD4, which interacts with sst2 and disrupts its signaling [18], may influence spontaneous or SSA-inhibited hormone secretion [19], as well as aberrant cell proliferation [18, 20], and has been proposed as a biomarker for increased risk of malignant behavior in certain tumors [18, 20, 21].

Another issue deserving further investigation in GEP-NETs concerns angiogenesis, since formation of new vessels from pre-existing vasculature is crucial for local invasion and metastatic spread of tumors. Molecules that exert important regulatory roles in angiogenesis in NETs include the vascular endothelial growth factor (VEGF), angiopoietins (Ang)-1 and -2, and the tyrosine kinase receptor Tie-2 (or Tek) family [22–24]. VEGF acts as a pro-angiogenic factor on vascular endothelium, inducing proliferation and new micro-vessel formation. Meanwhile, soluble angiopoietins, which are secreted by endothelial and epithelial cells in response to stress, hypoxia and inflammation, bind to Tie-2 to fulfill their actions. Specifically, Ang-1 promotes endothelial cell survival, and anti-inflammatory and anti-permeable effects [23–25], whilst Ang-2 causes vasculature regression or a marked pro-angiogenic effect if VEGF is present. Thus, the Ang/Tie2 system seems to play an important role in vascular network remodeling [26] and in the pathogenesis and progression of NETs [27]. Furthermore, a potential relationship of this angiopoietic system with SSA and their binding to ssts may also exist [28–33], although the precise roles of the different components of these systems and the potential interactions between them are insufficiently characterized.

Therefore, in this study, we aimed to determine the presence and potential functional roles of the novel truncated sst5 variants, and their association with the VEGF and Ang/Tie system, in human GEP-NETs.

## RESULTS

A total of 26 patients with GEP-NET were included in our study. Thirteen patients (50%) presented with pancreatic tumors (7 non-functional, 5 insulinomas and 1 ectopic Cushing) and the other 13 had gastrointestinal NETs. A total of 15 patients presented with metastasis, the majority of them in regional lymph nodes and/or liver. Pre-surgical CgA was determined in 22 patients, with a mean value of 19.7 ± 21.2 nmol/L (median 15.3 (0–77) nmol/L; reference range 0–6 nmol/L). Pre-surgical 5-hydroxy-indoleacetic acid was available in 8 patients, with a mean value of 17.2 ± 17.6 mg/24 h (median 7.8 (2–42) mg/24 h; reference range 2–10 mg/24 h). Immunoperoxidase staining for CgA and synaptophysin was positive in all tumor tissues. A Ki-67 immunoreactivity level > 2% was observed in 7 out of the 14 available samples (mean Ki 67 index 10.7 ± 23.3%; median 2.5 (2–90)%). A detailed summary of clinical and pathological features of the patients included in our study is shown in Table 1.

**Table 1:** Clinical, laboratory and pathological features of the 26 patients with gastro-entero-pancreatic neuroendocrine tumors

PATIENT	S	A	Tumor Type	Stage (ENETS)	WHO Grade	Metastasis (Location)	Presur-gery CgA*(nmol/L)	Presurgery Urinary 5-HIAA**(mg/24 h)	Presur-gery Octreo-scan	Postsur-gical SSA Treat-ment	Follow-up***
1	F	34	P	IIA	G2		ND	−	Negative	−	ND
2	F	41	P	IIIB	−	RLN	0	−	Negative	−	ND
3	F	67	P	I	−		0	−	−	−	ND
4	F	53	G	IIA	−		ND	−	−	−	ND
5	F	76	P	IV	−	RLN, L	5	4	Positive	+	RD
6	M	38	G	IV	−	RLN, L	0	42	Positive	+	RD
7	F	78	P	IV	−	RLN, L	33	−	Positive	+	RD
8^†^	M	58	P	IV	−	RLN, L, P	33	3	Positive	+	RD
9	F	78	G	IV	G1	RLN, L	6	−	Positive	+	RD
10	M	41	G	IV	G2		3	2	Negative	+	RD
11	F	71	P	I	G1		6	−	Negative	−	ND
12^^†^^	F	57	G	IV	G2	RLN, L, LM, B	59	32	Positive	+	RD
13	M	58	G	IV	G1	RLN, L	77	−	Positive	+	RD
14	F	73	P	IV	G2	L	18	−	Negative	+	RD
15	M	54	G	IV	G2	RLN, L	15	12	Negative	+	RD
16^†^	F	66	G	IIB	−		1	4	Negative	−	ND
17	F	44	G	IIB	G1		15	−	−	−	ND
18	M	58	G	IIIB	G1	RLN	1	−	−	−	ND
19	M	63	G	IV	G1	RLN, L	18	−	Positive	+	RD
20^^†^^	M	85	P	I	G3		8	−	−	−	ND
21	M	79	P	I	−		22	−	Positive	−	ND
22^†^	F	51	G	IV	−	RLN, L	46	−	Positive	+	RD
23	F	49	P	I	G1		22	−	−	−	ND
24	M	43	P	IIB	−		46	−	−	−	ND
25	F	44	P	I	G2		ND	−	−	−	ND
26	M	58	G	IV	−	RLN, L	ND	40	Positive	+	RD

Abbreviations: S: sex; A: age; CgA: chromogranin A; 5-HIAA: 5-hydroxy-indoleacetic acid; SSA: somatostatin analogues; M: male; F: female; P: pancreatic NET; G: gastrointestinal NET; RLN: regional lymph nodes; L: liver; P: peritoneum; LM: lung; NA: not available.

† patients died during follow-up.

* CgA Range: 1–6 nmol/L.

** 5-HIAA Range: 2–10 mg/24 h.

*** ND: non-residual disease, if there was a complete resection after surgery and no tumor relapse was evidenced during follow-up; RD: residual disease, in cases of tumor burden after surgery or relapse of disease during follow-up. Median follow-up was 87.5 months (19–214).

### SST receptors and the truncated variants are overexpressed in GEP-NETs

**Figure 1:**
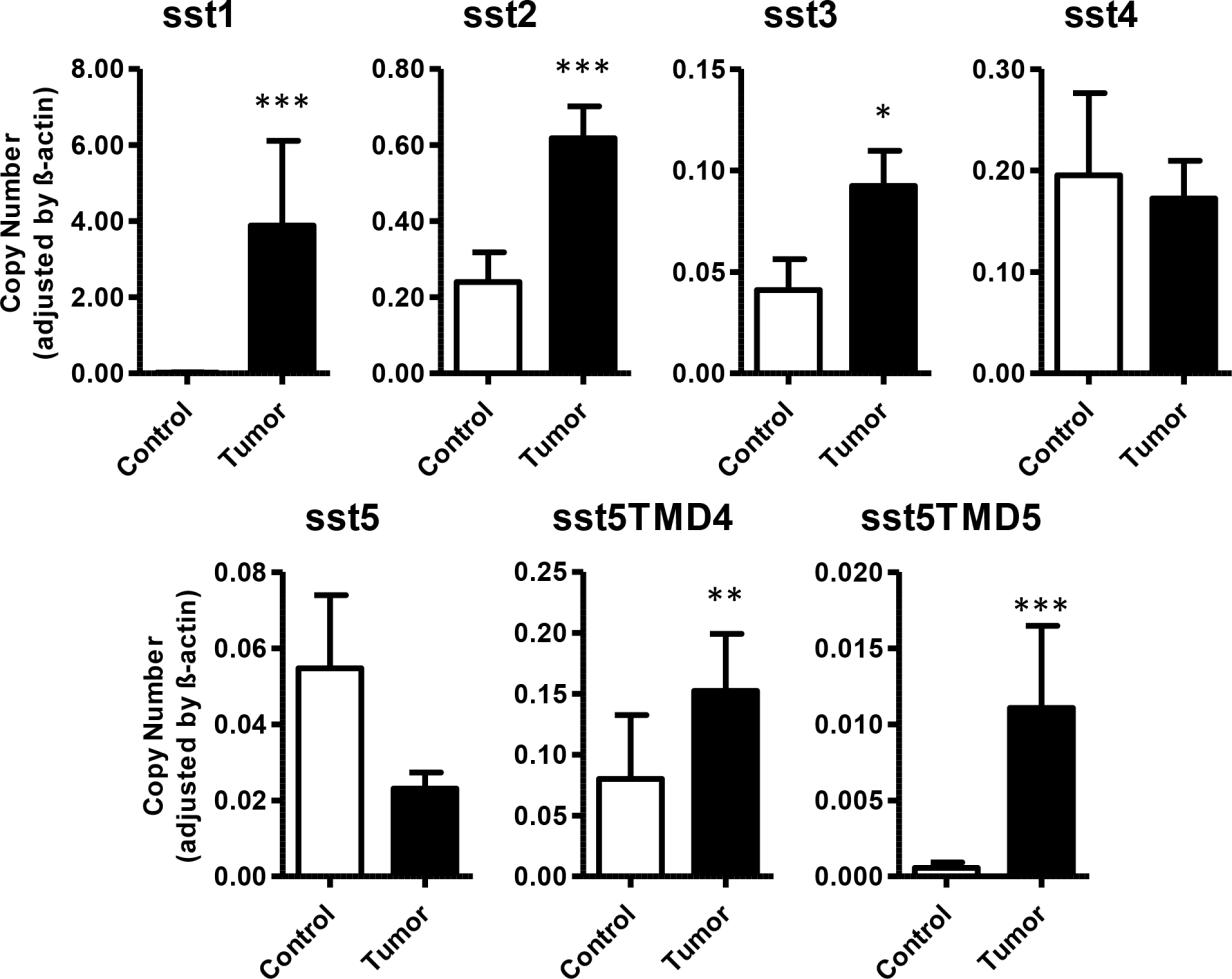
Expression of somatostatin receptors in GEP-NETs and adjacent non-tumor tissue Canonical (sst1–5) and truncated (sst5TMD4 and sst5TMD5) somatostatin receptors were measured by qPCR in a set of GEP-NETs, including primary and metastatic tissue. Values represent mean ± SEM of absolute mRNA values, adjusted by β-actin. Asterisks indicate significant differences between tumor and adjacent non-tumor tissues (*p*-values for *t*-test: *p < 0.05, **p < 0.01, ***p < 0.001).

qPCR in GEP-NET revealed expression of sst1 in 80.8% of cases, sst2 in 92.0%, sst3 in 56.0%, sst4 in 68.0%, and sst5 in 68.0%. Receptor subtype sst1 exhibited the highest expression in GEP-NET, followed by sst2 > sst4 > sst3. A significant increase in expression levels of sst1, sst2 and sst3 was observed in tumor tissues in comparison to adjacent non-tumor tissues (3.88 ± 2.23 vs. 0.02 ± 0.01; 0.62 ± 0.08 vs. 0.24 ± 0.08; and 0.09 ± 0.02 vs. 0.04 ± 0.02, respectively). However, no significant differences were observed in the expression of sst4 and sst5 between tumor and non-tumor samples (Figure 1). Interestingly, expression of the truncated subtypes sst5TMD4 and sst5TMD5 was detected in 25 and 19 cases, respectively, out of the 26 tumor samples evaluated (96.2% and 73.1%, respectively), whilst analysis of these receptors in adjacent, non-tumor/control tissues evidenced detectable expression in only 65.5% and 17.2% of cases, respectively (Table 2). Moreover, qPCR revealed an increased expression in tumor tissues in comparison to normal tissues (0.15 ± 0.05 vs. 0.08 ± 0.05, *p* < 0.01 for sst5MD4, and 0.011 ± 0.005 vs. 0.0006 ± 0.0004, *p* < 0.001 for sst5TMD5) (Figure 1). No statistical differences were found between normal tissue and adjacent “normal” tissue in the vicinity of the NET (Supplementary Figure 1). However, it is worth noticing that three of these adjacent non-tumor tissues (two of which were samples from liver metastases) had a high expression (outliers by Tukey's method) of the truncated variants. In agreement with this finding, immunohistochemical analysis of serial sections of normal (healthy) pancreas samples demonstrated that normal pancreatic islets (stained for CgA) did not show an evident sst5TMD4 specific staining (Supplementary Figure 2).

**Table 2:** Number of samples (%) in which somatostatin receptors were detected

	Tumor tissue	Adjacent non-tumor/control tissue
**sst1**	21/26 (80.8)	14/28 (50.0)
**sst2**	23/26 (92.0)	17/28 (60.7)
**sst3**	14/25 (56.0)	12/26 (46.2)
**sst4**	17/25 (68.0)	14/26 (53.8)
**sst5**	17/25 (68.0)	13/26 (50.0)
**sst5TMD4**	25/26 (96.2)	19/29 (65.5)
**sst5TMD5**	19/26 (73.1)	5/29 (17.2)
**SST**	22/26 (84.6)	19/26 (61.5)
**CORT**	16/24 (66.7)	14/25 (56.0)

Expression of SST and CORT was detected in 66.7% and 56.0% respectively (Table 1) but no significant differences in their expression were found between tumor and non-tumor samples (Supplementary Figure 3).

### sst5TMD4 is associated to enhanced malignancy features in patients with GEP-NETs and transfected cell lines

**Figure 2:**
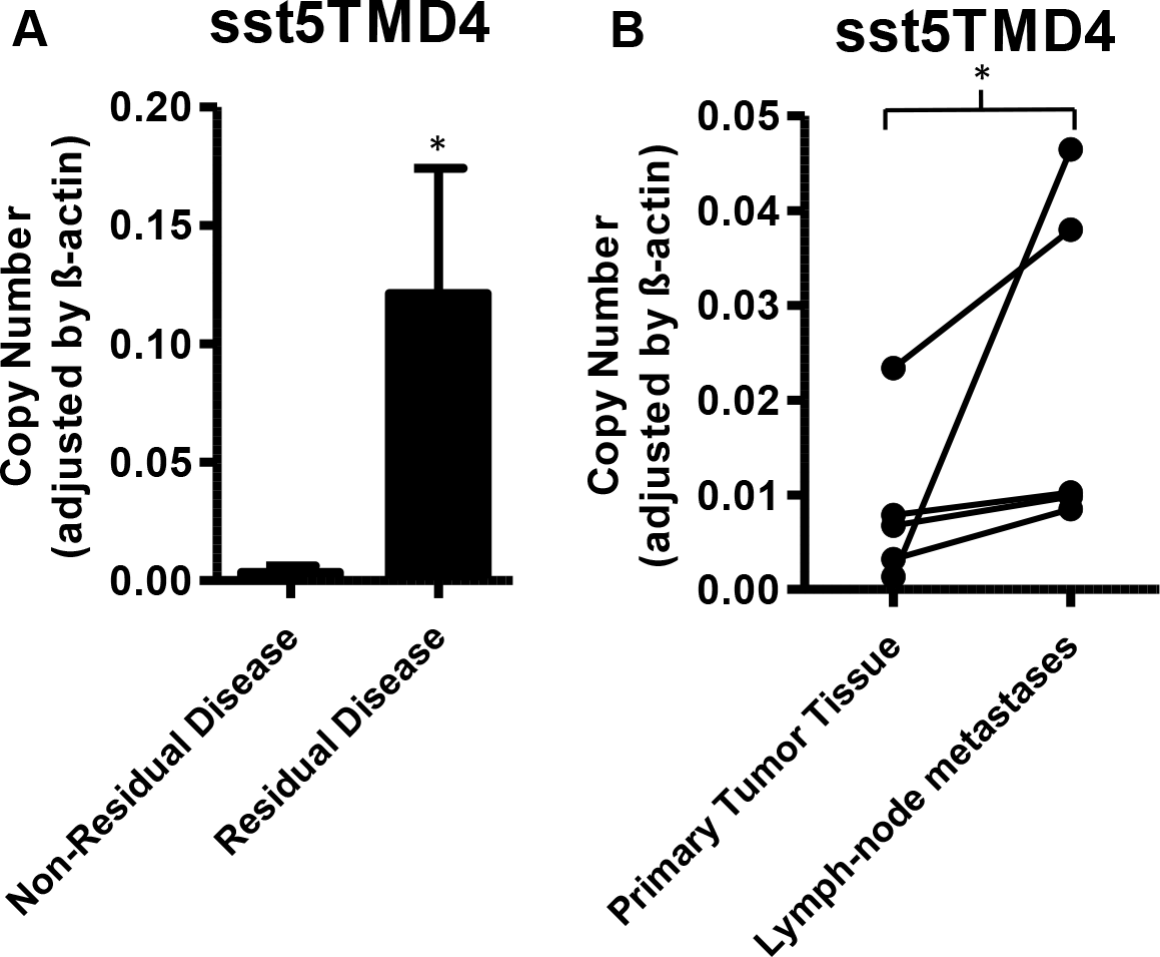
Expression of sst5TMD4 is associated to increased malignancy in patients with GEP-NETs (**A**) mRNA expression levels of sst5TMD4 in tumor samples of gastrointestinal origin. Patients were classified according to disease status in non-residual and residual disease. Values represent mean ± SEM of absolute mRNA values, adjusted by β-actin. (**B**): Paired analysis of sst5TMD4 expression in primary tumor tissue and lymph-node metastases of the same patients. Absolute mRNA level values were determined by qPCR and adjusted by β-actin. 1 was an intestinal tumor and 4 were pancreatic. Asterisks indicate significant differences (*p*-values for *t*-test: *p < 0.05).

Tumor tissues from gastrointestinal origin from patients with residual disease analyzed by qPCR exhibited higher expression of sst5TMD4, compared to those tumors from patients with non-residual disease (Figure 2A). However, no significant differences were found in tumors from pancreatic origin. Furthermore, a comparative analysis of the sst subtypes and their variants in paired biopsies from primary- and metastatic-site tumor tissues from the same patients revealed an increased expression of sst5TMD4 in lymph-node metastases, in comparison to its original corresponding primary tumor (Figure 2B). In contrast, there was no difference in sst receptor expression between distant metastases and their corresponding primary tumor (*p* > 0.05; 5 pairs analyzed).

**Figure 3:**
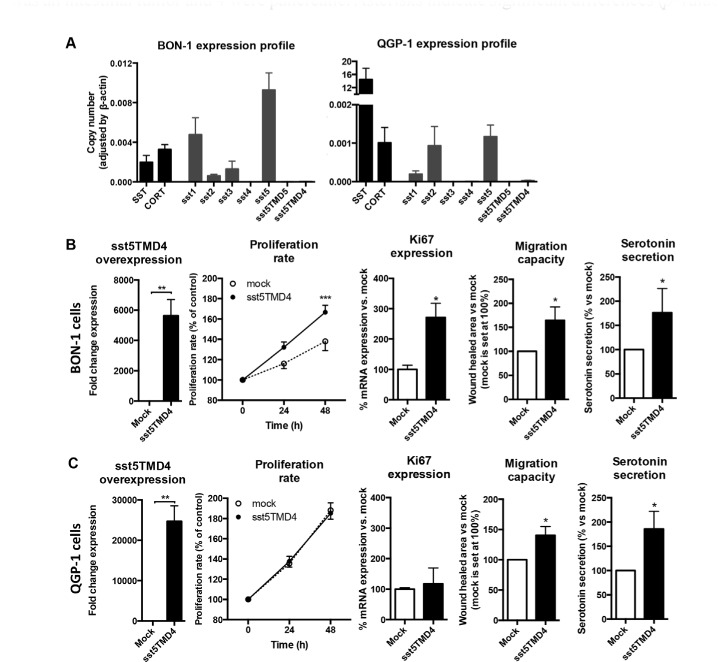
The presence of sst5TMD4 is associated to enhanced malignant features in NET cell lines (**A**) Normalized levels of SST, CORT and sst expression in BON-1 NET cell lines and QGP-1 NET cell lines. Functional assays in sst5TMD4-transfected BON-1 cell lines (**B**) and QGP-1 cell lines (**C**) in comparison to mock cells. Panel order from left to right: sst5TMD4 overexpression; proliferation rate; Ki67 expression; migration capacity; and serotonin secretion. Values represent mean ± standard error of the mean. Asterisks indicate significant differences (*p*-values for *t*-test: *p < 0.05, **p < 0.01, ***p < 0.001).

Expression of SST, CORT and both canonical and truncated ssts was also analyzed in BON-1 and QGP-1 cell lines by qPCR, which are commonly accepted as useful models for NET cell studies (Figure 3). Consistent with its origin from a human somatostatinoma, expression of SST was elevated in QGP-1 cells in comparison to CORT, but this was not the case for BON-1 cells (Figure 3A). When analyzing the different subtype receptors, we observed that sst5 showed the highest expression in both cell lines, while BON-1 presented moderate levels of both sst1 and sst3 (Figure 3A). Interestingly, both cell lines exhibited similar low levels of sst2 (Figure 3A). However, it is worth noting that truncated receptors were not detectable in any of these cell lines (Figure 3A). Thus, to further assess the potential impact of sst5TMD4 on malignancy features in NETs, we induced its overexpression in QGP-1 and BON-1 cell lines by sst5TMD4-vector transfection. qPCR of transfected cells confirmed successful transfection in both cell lines, where a high number of mRNA copies of sst5TMD4 was detected (Figure 3B and 3C – first panel). Using these cells as a model, we observed that proliferation rate at 48 h was significantly higher in BON-1 sst5TMD4-transfected cells (Figure 3B – second panel) than in controls. In contrast, no such differences in the proliferation rate were found between mock and sst5TMD4-transfected QGP-1 cells (Figure 3C – second panel). In agreement with this finding, a significant increase in Ki67 expression was observed in BON-1 sst5TMD4-transfected cells, but not in QGP-1 cells (Figure 3B and 3C – third panel). Further functional assays revealed that the presence of sst5TMD4 induced similar changes in both cell lines regarding their aggressiveness, since we could observe that sst5TMD4-transfected cells, but not mock cells, exhibited higher migration capacity (Figure 3B and 3C – fourth panel). In addition, functional capacity of sst5TMD4 transfected cells was higher as an increased serotonin secretion in comparison to mock cells was found (Figure 3B and 3C – fifth panel).

### Expression of truncated receptors correlates with expression of angiogenic markers in patients with GEP-NET

**Figure 4:**
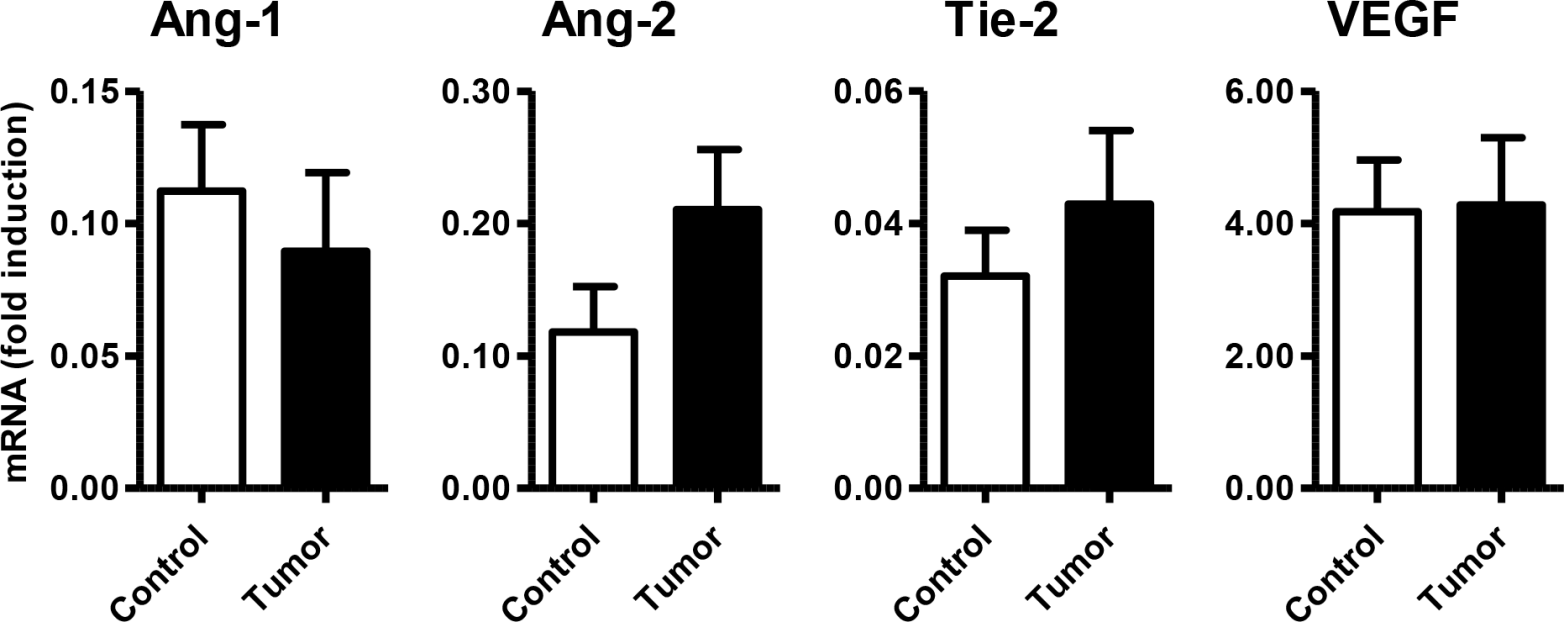
Expression of angiogenic molecules/markers in GEP-NETs and adjacent non-tumor tissue mRNA expression (fold induction) of Ang-1, Ang-2, Tie-2 and VEGF was measured by qPCR in a set of GEP-NETs, including primary and metastatic tissue. Results were normalized according to the value of β-actin. Values represent mean ± standard error of the mean.

**Figure 5:**
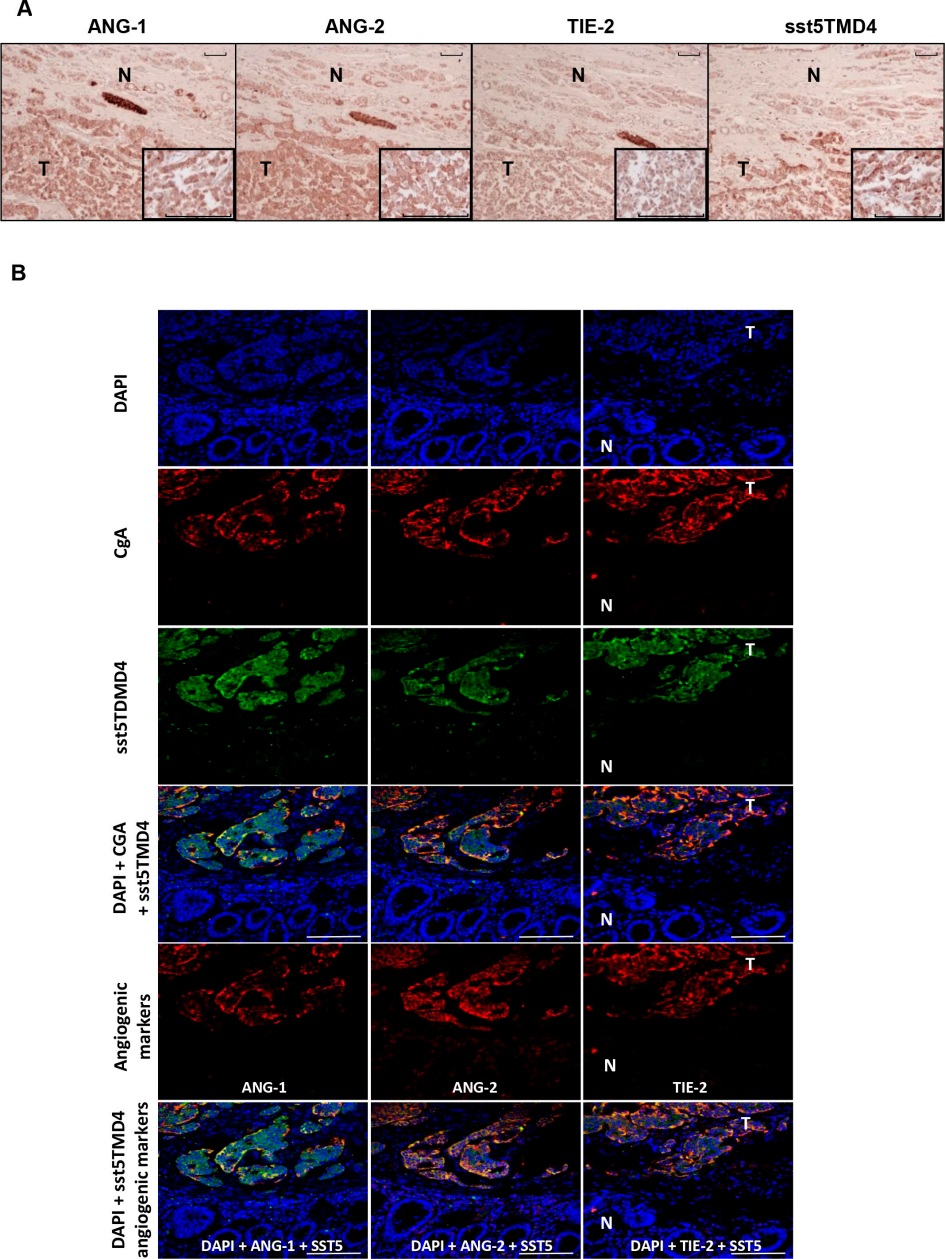
Expression of sst5TMD4 and co-localization with angiogenic marker in GEP-NET (**A**) Analysis of expression of angiogenic molecules and sst5TMD4 by specific serial immunohistochemistry in a pancreatic NET. Original magnification ×100 and ×400 (insets). N: normal tissue; T: tumor tissue. For specific immunostaining techniques see the “Materials and methods” section. (**B**) Expression of sst5TMD4 and angiogenic molecules by triple immunofluorescence in a gastrointestinal NET sample. Original magnification ×400. N: normal tissue; T: tumor tissue. For specific immunofluorescence techniques see the “Materials and methods” section. Scale bar for 100 μm is represented with a line for each Figure.

**Figure 6:**
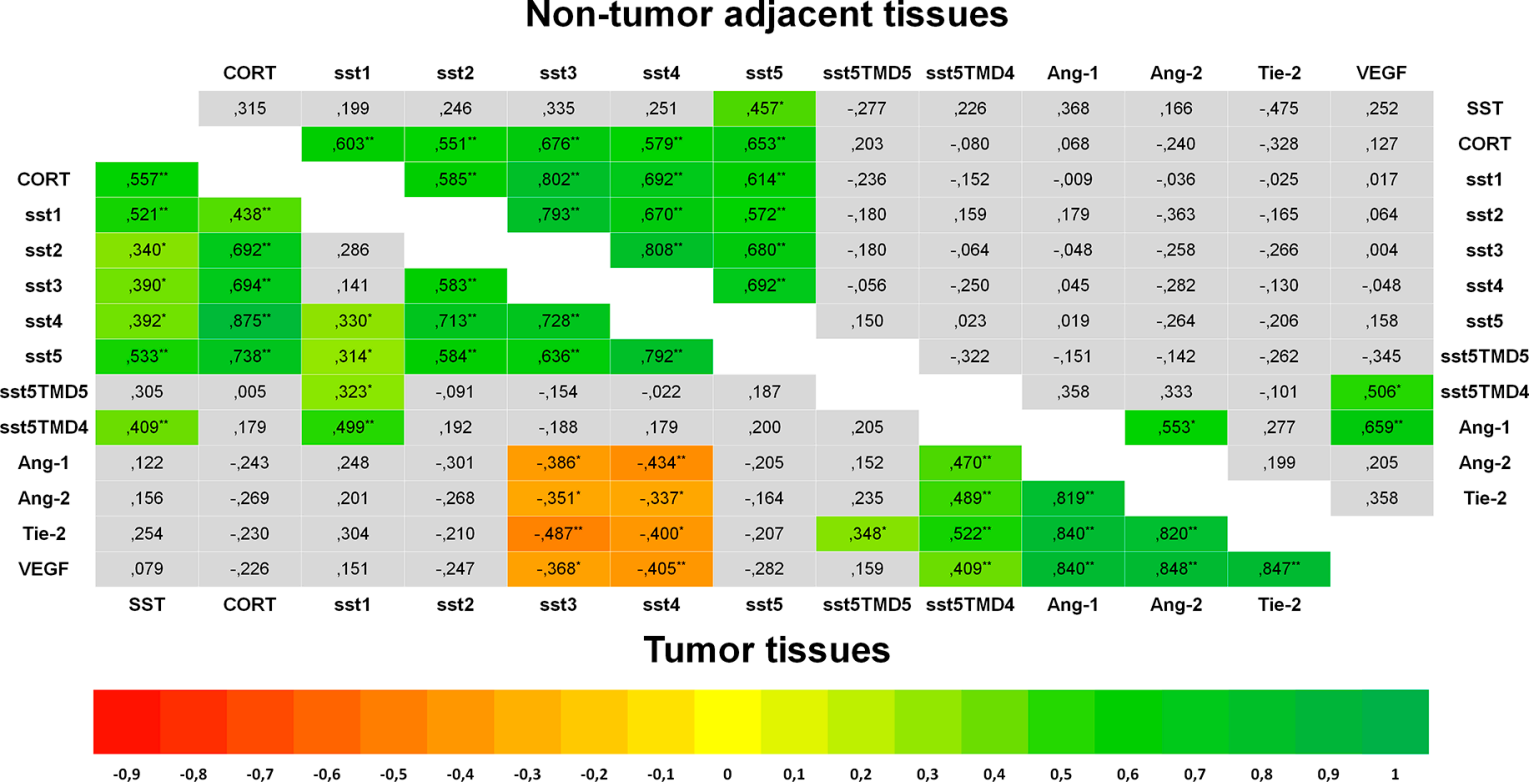
Heat-Maps for correlations between the expression of SST and angiogenesis systems (Spearman's Rho) Significant negative correlations are shown in red and significant positive correlations in green. Bottom left triangle shows correlations in tumor tissues and top-right hand triangle shows correlations in non-tumor adjacent tissues. In tumor samples, SST and CORT showed a significant positive correlation with sst1-5, and these subtypes were also positively correlated between themselves in a significant way. sst5TMD4 showed a positive correlation with SST and sst1, and sst5TMD5 with sst1. A positive correlation was found between the four angiogenic makers and a negative one between each one of them and sst3 and sst4. Ang-1, Ang-2, Tie-2 and VEGF were positively correlated with sst5MD4. Analyses of correlations in adjacent non-tumor tissues showed similar findings regarding CORT and sst1–5. However, SST was only correlated with sst5, and no correlation was observed between truncated receptors and the rest of subtypes. Asterisks mark significant *p*-values (*p < 0.05, **p < 0.01, two-sided).

Specific qPCR analysis for angiogenic markers confirmed the presence of Ang-1, Ang-2, Tie-2 and VEGF in GEP-NET (Figure 4). In addition, serial immunohistochemistry with specific antibodies in paraffin-embedded tissues from both pancreatic and gastrointestinal NETs, evidenced positivity for Ang-1, Ang-2 Tie-2 and sst5TMD4 in tumoral cells in serial sections (Figure 5A). IHS was evaluated in 16 tumor samples (14 primary and 2 metastatic). High expression of sst5TMD4 was found in 50% (7/14) of primary tissues and 100% (2/2) of metastatic tissues. Median IHS values were 100 (range 0–300) for sst5TMD4, 208 (70–300) for Ang-1, 185 (60–270) for Ang-2, and 189 (80–285) for Tie-2. These results were corroborated by triple immunofluorescence studies (Figure 5B). We observed co-expression of sst5TMD4 (Figure 5B lane 2 and 3) and CgA in neuroendocrine tumor cells, but not in adjacent non-tumor cells (Figure 5B lane 4). In addition, co-expression of sst5TMD4 and different angiogenic markers was also evidenced in neuroendocrine cells (Figure 5B lane 6). Spearman's Rho analyses in tumor tissues revealed positive significant correlations between the four angiogenic markers, and a negative correlation of each one of them with receptor subtypes sst3 and sst4 (Figure 6). Furthermore, Ang-1, Ang2, Tie-2 and VEGF were all directly and significantly correlated with sst5TMD4, and Tie-2 showed this same relationship with sst5TMD5 (Figure 6 – bottom left). On the contrary, however, in the few samples of adjacent non-tumor tissue in which sst5TMD4 and VEGF were detected, positive correlations were only observed for Ang-1 and Ang-2, and VEGF with Ang-1 and sst5TMD4 (Figure 6 – top right). In view of these results, the expression of these pro-angiogenic factors and the secretion of VEGF were determined in mock and sst5TMD4-transfected BON-1 and QGP-1 cells. As shown in Supplementary Figure 4, sst5TMD4 overexpression in both cell lines did not increase the expression of pro-angiogenic factors (VEGF, Ang-1 or Ang-2) or the release of VEGF to the culture media.

## DISCUSSION

In this study, we have investigated the expression pattern of somatostatin receptors, particularly their truncated sst5 variants, as well as key markers of angiogenesis in a group of patients with GEP-NET. Additionally, we have evaluated their potential functional relationship. To our knowledge, this is the first time in which this has been thoroughly analyzed in a relatively large series of samples, especially when considering the limited prevalence of this uncommon heterogeneous group of neoplasms.

In the present series, we observed a differential sst expression pattern in tumor samples compared to their corresponding adjacent non-tumor tissues. Specifically, sst1–3 were significantly overexpressed in tumor samples in comparison to adjacent non-tumoral tissue. Our observations confirm previous reports and add further information regarding canonical somatostatin receptors (sst1–5) [9, 42, 43]. On this basis, it is worth emphasizing that identifying the potential influence of sst5TMD4 and sst5TMD5 in GEP-NET may entail important clinical consequences, especially given the fact that SSA treatment is virtually standardized in medical practice following non-curative surgery of GEP-NET [37].

A relevant finding of our study was that these truncated variants of sst5 were associated to enhanced malignancy features. Firstly, we found an association between the expression of these canonic receptors and disease status, with sst5TMD4 being higher in those tumors from patients with gastrointestinal NETs and residual disease, compared to those with non-residual disease after surgery. Residual disease includes invasive and/or disseminated tumors, either because they were not resectable by surgery or due to tumor recurrence after a prior complete resection. Therefore, our findings suggest a possible link between sst5TMD4 and worse clinical outcome. Secondly, paired analyses identified an increased expression of sst5TMD4 in metastatic lymph nodes in comparison to their corresponding primary tumors. This suggests that primary tumors expressing sst5TMD4 could potentially develop lymph node metastasis and/or local progression more frequently. Indeed, functional assays with sst5TMD4-trasfected BON-1 cells of carcinoid origin evidenced an increased proliferation rate and Ki67 expression. Interestingly, although sst5TMD4-trasfected QGP-1 cells did not mimic these results, presumably due to their different nature (i.e. the considerable SST expression in the latter), both sst5TMD4-trasfected NET cell lines exhibited higher migration capacity. Moreover, transfected cells preserved their ability to secrete serotonin and, in fact, serotonin levels were significantly increased in these cells.

In a previous study, we demonstrated that transfection of sst5TMD4 in MCF-7 cells, a model for breast cancer, increased expression of Arp-2/3 (Actin-related proteins) [18], a complex that plays a major role in the regulation of actin filaments, and is associated to the enhanced ability of cancer cells to invade [44]. Also, in this same model, we found that cells with endogenous expression of sst5TMD4 showed higher levels of p-Akt and p-ERK1/2, two kinases that activate signal transduction pathways involved in proliferation, migration and phenotype transformation in cancer cells. Furthermore, we have recently reported sst5TMD4 overexpression in thyroid cancer, both medullary and non-medullary [20, 21]. sst5TMD4 overexpression in TT cells confers a greater growth capacity, modifies the cell's phenotype, decreases E-cadherin and phosphorylated β-catenin levels, increases vimentin, total β-catenin and phosphorylated GSK3B levels, and confers a greater invasion capacity [21].

There are other profoundly complex molecular pathways involved in the pathogenesis of GEP-NETs. For instance, dysregulation of the phosphoinositide 3-kinase (PI3K)-Akt-mTOR pathway [45], or aberrant signaling through G protein-coupled receptors like ssts, may lead to modulation of several key enzymes, including adenylyl cyclase, phosphotyrosine phosphatases (PTPs) and MAPKs (mitogen activated kinases) [46–48]. In this complex molecular scenario, it seems biologically plausible that sst5TMD4 plays a relevant role in the specific setting of GEP-NET, since, despite being a truncated receptor variant, it can influence and interfere with subsequent regulation of several molecular pathways. Although further investigations regarding these complex molecular relationships deem necessary to clarify this issue, sst5TMD4 may be indeed associated to tumor progression and possibly a worse clinical outcome in GEP-NET.

Given the vascular nature of NETs, which has been demonstrated in different experimental models [27, 49], angiogenesis is likely to play a relevant role in the pathogenesis and progression of GEP-NET. In this regard, we confirmed the presence of key angiogenic markers, Ang-1, Ang-2, Tie-2 and VEGF, in tumor samples from patients with GEP-NET and in transfected cell lines. Further, tumor expression of these markers showed a positive correlation between them, and a negative correlation with subtypes sst3 and sst4, in agreement with the above-mentioned studies. Interestingly, in addition, we report another novel finding: expression of the truncated variant sst5TMD4 was positively correlated with all four angiogenic markers. Immunohistochemical and immunofluorescence studies confirmed co-expression of angiogenic markers and of sst5TMD4 in CgA^+^ tumor cells. Furthermore, immunohistochemical analysis of normal (healthy) pancreatic tissue did not evidence a detectable expression of sst5TMD4 in CgA^+^ islet cells. These results further support the hypothesis of the potential interference of this variant in the putative signaling pathway of canonic non-truncated sst subtypes, as it has been proposed in earlier studies [18], as well as with angiogenic molecules, as also suggested by our present data. SSAs are known to exert an anti-angiogenic effect through their interaction with ssts, by inhibiting production and secretion of many angiogenic factors [10, 11]. In this context, previous studies have reported that signaling through sst3 down-regulates VEGF production [29], that sst1 signaling inhibits endothelial proliferation, migration and neovascularization [32, 50], and that, through sst1-3, the endothelial nitric oxide synthase (NOS) is inhibited [29, 30]. Moreover, sst2 expression has been shown to have an anti-angiogenic role in animal models of hypoxia [51], suggesting an active interplay between the somatostatin-signaling network and sustained angiogenesis. Also, somatostatin secretion is known to negatively influence VEGF production [52], and *in vitro* experiments have shown that the administration of the SSA octreotide can antagonize the hypoxia inducible factor 1a (Hif-1a) transcriptional activity in NET cells [8]. In line with these reports, our present findings favor the hypothesis that sst5TMD4 could play an important role in the complex molecular network of vascularization signaling. Specifically, if we take into account the fact that sst5TMD4 interacts with sst2 [18, 19], the subsequent decreased and/or abnormal activation of sst2-associated transduction signaling pathways would plausibly lead to the development of increased vascularization, by reducing its usual antiangiogenic effect.

Although we acknowledge the limitations of our study regarding the number of samples evaluated and its retrospective observational nature, we would like to highlight the fact that, to our knowledge, this is one of the largest GEP-NET series in which such a thorough qPCR analysis of sst subtypes, including the truncated variants, and angiogenesis-related molecular markers has been performed. Besides, we should also bear in mind that analysis of surrounding non-tumoral tissue, adjacent to each corresponding tumor, is not the ideal method for comparison with tumor tissue in the case of NETs, as it has been widely recognized in this research field; this should be considered only as a reference tissue, rather than as a genuine control, but it serves for the purpose of investigations in this topic.

In conclusion, we report for the first time a significant overexpression of the truncated variants sst5TMD4 and sst5TMD5 in GEP-NET, as well as increased levels of sst5TMD4 in patients with residual gastrointestinal NET and in lymph-node metastases, in relation to its corresponding primary tumor tissue. sst5TMD4 was associated to an enhanced proliferation rate, migration capacity and serotonin secretion in NET model cell lines, and to a relationship with angiogenic markers in tumor tissues. Taken together, our results suggest that the truncated variant sst5TMD4 could be involved in local progression and worsen prognosis in GEP-NET. Results of our various analyses contribute to a better characterization and knowledge of GEP-NET, and allow more accurate and evidence-based prognostic estimations. Further long-term and prospective studies deem necessary to better understand the relevance of these ssts subtypes, specially the truncated sst5 variants, in the pathophysiology and clinical/prognostic features of GEP-NET.

## MATERIALS AND METHODS

### Study population

We reviewed 26 consecutive patients (15, 57.7% females, mean age 58.4 ± 14.4 years old) with GEP-NET who underwent surgery at our center from 2001 to 2009. All patients were carefully screened for the presence of other malignancies, and special attention was paid for an association with genetic syndromes (multiple endocrine neoplasia type 1, von Hippel-Lindau syndrome, tuberosclerosis and neurofibromatosis syndromes). Only one patient carried a mutation for multiple endocrine neoplasia type 1.

Data regarding physical examination, medical history, and laboratory work-up were obtained from routine visits using information available in clinical records. Patients were classified according to the ENETS and WHO criteria (tumor site and size, Ki67, mitotic rate and metastases) [34, 35]. Additionally, according to histopathology findings, all well-differentiated neoplasms were classified as NETs and graded G1 (Ki67 < 2%) or G2 (Ki67 2–20%), and all poorly differentiated neoplasms were designated as neuroendocrine carcinomas (NECs) and graded G3 (Ki67 > 20%) [36].

Patients were managed following current recommendations and guidelines [37]. Elective surgery was the first option of treatment in all cases and adjuvant therapy with SSAs was administered if evidence of residual disease was observed. Follow-up evaluation classified patients into two categories according to their clinical status: 1) non-residual disease, if a complete resection after surgery had been achieved and no tumor relapse was evidenced during follow-up and 2) residual disease, in cases of tumor burden after surgery or relapse of disease during follow-up. The median of follow-up was 87.5 months (19–214).

The study was approved by the local Ethical Committee and conducted in accordance to the principles of the Declaration of Helsinki, and all patients signed a written informed consent before inclusion.

### Samples

A total of 72 formalin-fixed paraffin-embedded tissues were evaluated. Of these, 42 were proper tumor samples with pathological diagnosis of NET (26 from the primary site and 16 from a metastatic site), and 30 corresponded to normal tissues (26 samples form adjacent non-tumor regions and 4 normal control tissues that had been obtained from patients undergoing pancreatic, intestinal or hepatic resection), which were used as qRT-PCR negative controls. Additionally, three normal pancreatic tissues were used as immunohistochemistry negative controls. All samples were taken and managed in accordance with regulations and approval of the local Institutional Review Board.

A thorough review of hematoxylin and eosin (H&E) sections by a board-certified endocrine pathologist (MA) was carried out to ensure identification of relevant and representative areas of tumor and non-tumor tissues to proceed to RNA extraction. Simultaneously, immunohistochemical staining was performed in paraffin embedded blocks by the avidin-biotin peroxidase complex (ABC) method, using anti-human CgA antiserum (Biogenex Laboratories, San Ramon, CA, USA), synaptophysin, and proliferation-related Ki-67 antigen (Dako Cytomation Denmark A/S, Copenhagen, Denmark); as well as insulin, SST, glucagon and gastrin. Tumors were then classified following current guidelines [36].

### Cell culture

In order to provide a biological basis for functional assays, previously validated NET cell lines were cultured. Specifically, carcinoid BON1 cells [38] and somatostatinoma-derived QGP1 cells [39] were used. BON-1 was cultured in Dulbecco's Modified Eagles Medium (DMEM-F12; Life Technologies, Barcelona, Spain) supplemented with 10% fetal bovine serum (FBS; Sigma-Aldrich, Madrid, Spain), 1% glutamine (Sigma-Aldrich) and 0.2% antibiotic (Gentamicin/Amphotericin B; Life Technologies). QGP-1 was maintained in RPMI 1640 (Lonza, Basel, Switzerland), supplemented with 10% FBS, 1% glutamine and 0.2% antibiotic. Both cell lines were grown at 37°C, in a humidified atmosphere with 5.0% CO2.

### RNA isolation and retrotranscription

Total RNA was isolated using TRIzol Reagent in the case of cell lines (Life Technologies) following the manufacturer's instructions and treated with DNase (Promega, Barcelona, Spain). Total RNA extraction from paraffin samples was performed using RNeasy FFPE Kit (Qiagen, Limburg, Netherlands) according to the manufacturer's protocol. The amount of RNA recovered (before and after DNase treatment) was determined using the NanoDrop2000 spectrophotometer (Thermo Scientific, Wilmington, NC, USA). Quality of RNA extracted was assessed by the same system using the Absorbance Ratio A260/280 and A260/230, requiring a minimum of 1.8 on both to perform qPCR. One microgram of RNA was reverse transcribed to cDNA using random hexamer primers [First Strand Synthesis (Thermo Scientific)].

### Quantitative real time PCR (qPCR)

qPCR reactions were performed using the Brilliant III SYBR Green Master Mix (Stratagene, La Jolla, CA, USA) in the Stratagene Mx3000p system for sst1-5, sst5TMD4, sst5TMD5 and their ligands somatostatin (SST) and cortistatin (CORT). For each reaction, 10 μl of master mix, 0.3 μl of each primer, 8.4 μl of distilled H2O and 1 μl of cDNA (50 ng) in a 20 μl total volume were mixed. Specifically, the program consisted of the following steps: (1) 95°C for 3 min, (2) 40 cycles of denaturing (95°C for 20 sec) and annealing/extension (61°C for 20 sec) and (3) a last cycle where final PCR products were subjected to graded temperature-dependent dissociation (55°C to 95°C, increasing 0.5°C/30 sec) to verify that only one product was amplified. Specific primers (Supplementary Table 1) for human transcripts were designed with Primer3 software and StepOne™ Real-Time PCR System software v2.3 (Applied Biosystems^®^, Foster City, CA, USA). Results were validated as previously reported [40]. Samples were run in the same plate against a standard curve to estimate absolute mRNA copy number (1, 10^1^, 10^2^, 10^3^, 10^4^, 10^5^, and 10^6^ copies of synthetic cDNA template for each transcript), and a No-RT sample as a negative control. Normalization of all genes was done according to the value of beta-actin housekeeping gene. Results were presented as total copy number, adjusted for beta-actin.

qPCR reactions for Ang-1, Ang-2, Tie-2 and VEGF were performed using LightCycler Detection System (Roche Diagnostics, Madrid, Spain) and LightCycler FastStart DNA Master SYBR Green I kit (Roche Diagnostics).

### Immunohistochemistry and immunofluorescence studies

Immunohistochemistry was performed on formalin-fixed paraffin-embedded sections. Tissue sections were dewaxed with xylene and rehydrated using decreasing concentrations of alcohol. Antigen retrieval was obtained by incubation in commercial 10 mM citrate solution (pH 6.0; Master Diagnostica, Granada, Spain) using a microwave oven for 15 min at maximum power (700 W). Prior to immunostaining, slides were cooled down to room temperature and endogenous peroxidase activity was removed by incubation with a peroxidase blocking solution (Methanol 3% H_2_O_2_) for 25 min, under gentle stirring. Then, sections were incubated overnight at 4°C with rabbit polyclonal anti-sst5TMD4 antibody [12], goat polyclonal anti-Ang-1 (Cat. No. AF923, R&D Systems, Minneapolis, MN, USA), goat polyclonal anti-Ang-2 (Cat. No. AF623, R&D Systems), goat polyclonal anti-Tie-2 (Cat. No. AF313, R&D Systems) and mouse monoclonal anti-CgA antibody (NBP2-33198AF488, Alexa Fluor 488, Novusbio Littleton, CO, USA) subsequently incubated with the appropriate HRP-conjugated secondary antibodies (Envision system, Dako, Barcelona, Spain). Finally, sections were developed with 3.3′-diaminobenzidine (Envision system 2-Solution DAB Kit), counterstained with Carazzi's hematoxylin, dehydrated in alcohol, cleared with xylene, and mounted. Negative control reactions were performed by omitting the primary antibody from the dilution buffer. This resulted in a completed absence of staining in all cases. Sections were analyzed in a Nikon Eclipse E400 optical microscope (Nikon, Japan).

A single histopathologist (MA), blinded to clinical data, scored all IHQ and IF cases. Tissue samples were scored manually using the immunohistochemical score method (IHS) proposed by Pinato et al. [41]. Specifically, for each sample, an IHS from 0 to 300 was assigned, based on the multiplication of the percentage of cells showing immunohistochemical expression (0–100) and the intensity of the signal (graded 1–3) in a minimum of 100 cells per slide. Every score was then re-assessed individually, and the mean of three readings was calculated.

Next, immunofluorescence techniques were performed to examine the pattern of staining and co-expression of sst5TMD4, angiogenic markers and CgA. Tissue sections were dewaxed and antigen retrieval was performed as stated before, were blocked with normal human IgG and incubated with rabbit polyclonal anti-sst5TMD4 antibody, goat polyclonal anti-Ang-1 (Cat. No. AF923, R&D Systems, Minneapolis, MN, USA), goat polyclonal anti-Ang-2 (Cat. No. AF623, R&D Systems), goat polyclonal anti-Tie-2 (Cat. No. AF313, R&D Systems) and mouse monoclonal anti-CgA antibody (NBP2-33198AF488, Alexa Fluor 488, Novusbio, Littleton, CO, USA) for one hour, followed by the proper secondary AlexaFluor 647 donkey-anti-goat (DAG) (Applied Biosystems, Carlsbad, CA, USA) and a biotinylated Donkey Anti Rabbit was used with Streptavidin-RhoX 568 (Applied Biosystems, Carlsbad, CA, USA). Hoechst 33342 dye was used for cell nuclei staining, and sections were analyzed in a Leica TCS-SP5 confocal microscope (Leica Microsystems, Wetzlar, Germany).

### Stable transfection of the truncated receptor sst5TMD4

BON1 and QGP1 cell lines were stably transfected with sst5TMD4-containing pCDNA3.1+ vector (Life Technologies) and selected as previously reported [18]. Specifically, BON1 and QGP1 cells were seeded in 6-well culture plates and transfected with sst5TMD4 o empty (mock) vectors using Lipofectamine 2000 Transfection Reagent (Life Technologies) following manufacturer's instructions and selected by geneticin treatment (Gibco, Barcelona, Spain). Stably-transfected cells were characterized by qPCR.

### Alamar blue proliferation assay

Cell proliferation of transfected cell lines was measured by the Alamar Blue fluorescent assay (Life Technologies). Briefly, cells were seeded in 96-well plates at a density of 3,000-5,000/well. Basal, 24 h and 48 h cell viability was determined by measurement of fluorescent signal exciting at 560 nm and reading at 590 nm (Flex Station 3; Molecular Devices) at 570 nm. Specifically, the day of measurement, cells were incubated for 3 h in 10% alamar blue/ serum free-media, and then, alamar reduction was measured. Results are expressed as percentage vs. control (mock transfected cells). Medium was replaced by fresh medium immediately after each measurement. In all instances, cells were seeded per quadruplicate and all assays were repeated a minimum of four times.

### Migration capacity assay

The ability of mock and sst5TMD4 stably transfected cells to migrate was evaluated by wound healing technique. Briefly, stable cells were plated at sub-confluence in 6 well plates. Confluent cells were serum-starved for 24 h and after synchronization the wound was made using a 100 μl sterile pipette tip. Wells were rinsed in PBS and then cells were incubated for 24 h in FBS supplemented medium. Wound healing was calculated as the area of a rectangle centered in the picture 24 h after the wound vs. the area of the rectangle just after doing the wound. At least three experiments were performed in independent days, in which three random pictures along the wound were acquired per well.

### *In vitro* secretion assay and angiogenic marker expression

To determine serotonin and VEGF secretion, mock and sst5TMD4 stably transfected BON1 and QGP-1 cells were seeded in 12 wells plates. At 70% confluence, cells were serum starved and after 24 h incubation, media were collected and stored at −20°C until measurements. Secretion of serotonin was detected using a serotonin ELISA kit (ALPCO, Salem, NH, USA) and VEGF secretion by using a VEGF ELISA Kit (Thermo Scientific). Results were expressed as percentage of serotonin or VEGF secretion vs. control (mock transfected cells). In addition VEGF, Ang-1, Ang-2 and Tie-2 were measured in cell lines by qRT-PCR. At least four experiments were performed in independent days, where the cells were seeded per duplicate.

### Statistical analysis

Descriptive results were expressed as mean ± standard deviation (SD), mean ± standard error of the mean (SEM), or median and minimum/maximum, as appropriate. Spearman's bivariate correlations were performed for all quantitative variables and differences between groups were compared using analysis of variance (U-Mann Whitney or Kruskal-Wallis ANOVA, as appropriate). Comparison between related variables was performed using Wilcoxon sum rank test. Samples from all groups within an experiment were processed at the same time. The *p*-values were two-sided and statistical significance was considered when *p* < 0.05, data is presented making specification for *p* < 0.05, *p* < 0.01 and *p* < 0.001. All statistical analyses were performed using SPSS version 20.0 (IBM SPSS Statistics Inc., Chicago, IL) and GraphPad version 5.0 (GraphPad Software, La Jolla, CA).

## SUPPLEMENTARY MATERIALS TABLE AND FIGURES


