# Supplementary material for: Presence of sst5TMD4, a truncated splice variant of the somatostatin receptor subtype 5, is associated to features of increased aggressiveness in pancreatic neuroendocrine tumors
Source: Oncotarget. 2015 Dec 11;7(6):6593–608. doi: 10.18632/oncotarget.6565 (PMC4872735; doi:10.18632/oncotarget.6565)
Supplement: Supplementary file 1 [file oncotarget-07-6593-s001.pdf]

Presence of sst5TMD4, a truncated splice variant of the somatostatin receptor subtype 5, is associated to features of increased aggressiveness in pancreatic neuroendocrine tumors

Supplementary Materials

Supplementry Table 1: Primer sequences, product sizes and GeneBank accession numbers

Gene	Sense	Antisense	Product lenght (bp)	GeneBank Accession number
**SST**	AACCCAACCAGACGGAGAA	TAGCCGGGTTTGAGTTAGCA	111	BC032625
**CST**	CTCCAGTCAGCCCACAAGAT	CAAGCGAGGAAAGTCAGGAG	173	NM001302
**sst1**	CACATTTCTCATGGGCTTCCT	ACAAACACCATCACCACCATC	165	BC035618
**sst2**	GGCATGTTTGACTTTGTGGTG	GTCTCATTCAGCCGGGATTT	185	NM001050
**sst3**	TGCCTTCTTTGGGCTCTACTT	ATCCTCCTCCTCAGTCTTCTCC	190	NM001051
**sst4**	CGTGGTCGTCTTTGTGCTCT	AAGGATCGGCGGAAGTTGT	174	BC069063
**sst5**	CTGGTGTTTGCGGGATGTT	GAAGCTCTGGCGGAAGTTGT	183	NM001053
**sst5TMD5**	GCGCCGTCTTCATCATCTAC	CAGGAAAAGCTGGTGTTTGG	159	DQ448303
**sst5TMD4**	TACCTGCAACCGTCTGCC	AGCCTGGGCCTTTCTCCT	98	DQ448304
**B-actin**	ACTCTTCCAGCCTTCCTTCCT	CAGTGATCTCCTTCTGCATCCT	176	NM001101

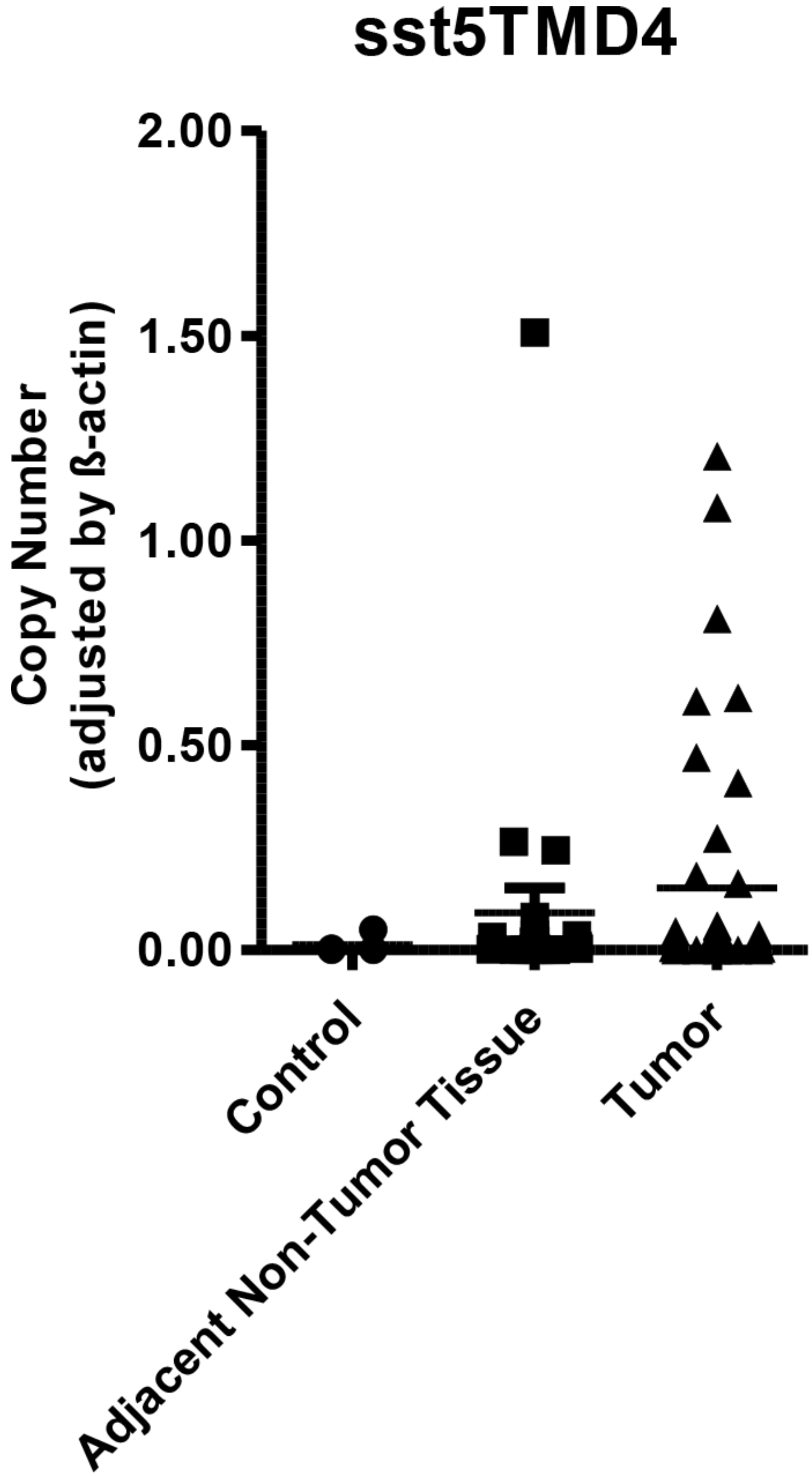

Supplementary Figure S1: Expression of sst5TMD4 in adjacent non-tumor tissue compared to normal tissues. sst5TMD4 receptors were measured by qPCR in a set of adjacent non-tumor tissues compared to normal tissues. Values (adjusted by β-actin) are represented by a dot-plot.

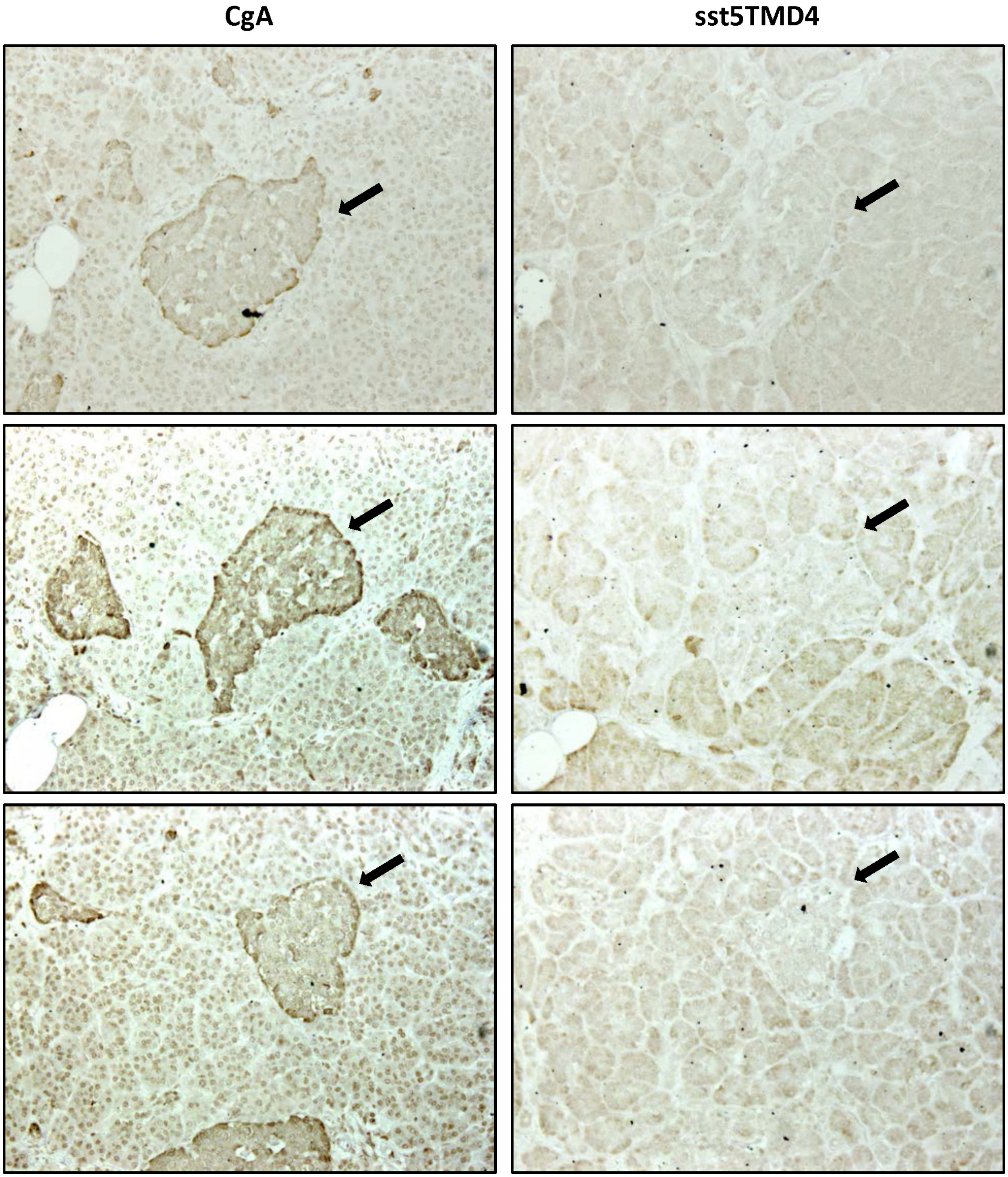

Supplementary Figure S2: Immunohistochemical staining of normal (healthy) pancreas for cromogranin A (CgA) and truncated sst5TMD4 receptor. Serial sections of normal (healthy) pancreas samples were stained for CgA and sst5TMD4 and representative images are depicted. Original magnification ×200. Arrows indicate pancreatic islets stained for CgA but lacking sst5TMD4 specific staining.

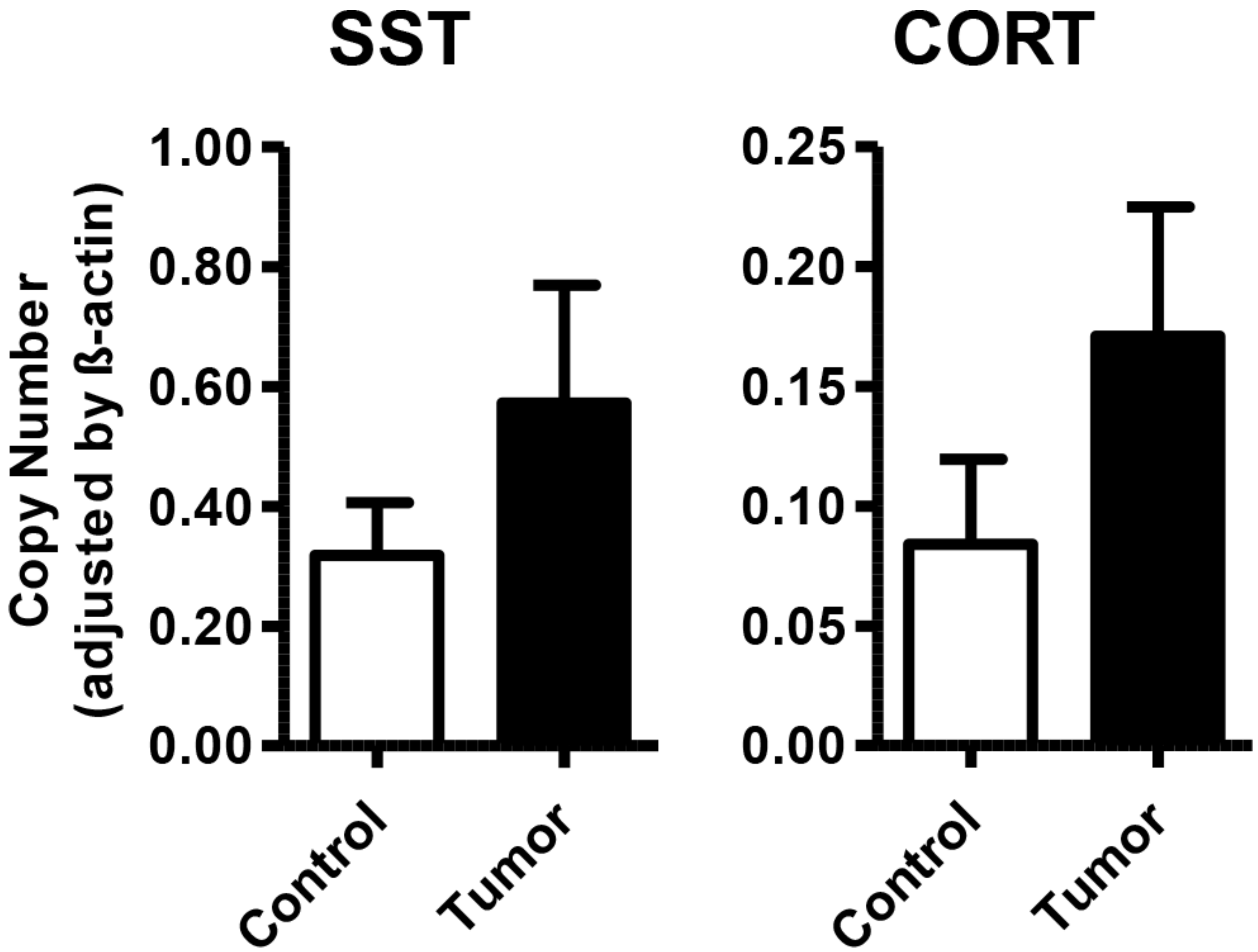

Supplementary Figure S3: Expression of SST and CORT in GEP-NETs and adjacent non-tumor tissue. SST and CORT were measured by qPCR in a set of GEP-NETs, including primary and metastatic tissue. Values represent mean ± SEM of absolute mRNA values, adjusted by β-actin.

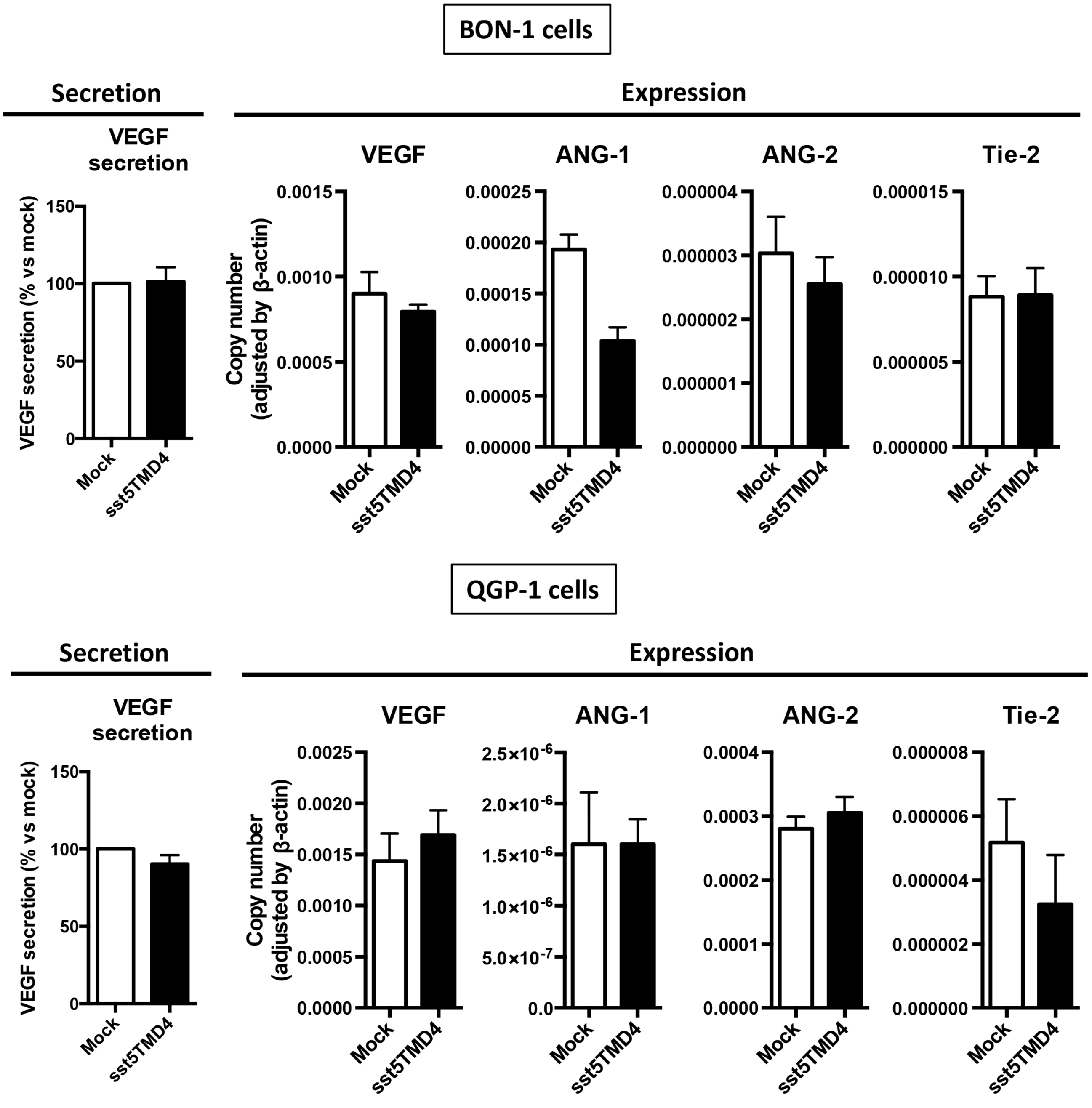

Supplementary Figure S4: Secretion of VEGF and expression of pro-angiogenic factors in NET derived cell lines. sst5TMD4 stably transfected BON-1 and QGP-1 cell lines were used to determine the effect of sst5TMD4 on VEGF secretion (by ELISA) and on the expression of pro-angiogenic factors (VEGF, ANG-1, ANG-2 and Tie-2) by qPCR. Values represent mean ± standard error of the mean.
